# Seven years of experience with treatment of benign paroxysmal positional vertigo with a mechanical rotational chair

**DOI:** 10.3389/fneur.2022.981216

**Published:** 2022-08-25

**Authors:** Dan Dupont Hougaard, Sebastian Hygum Valsted, Niels Henrik Bruun, Mathias Winther Bech, Michel Heide Talebnasab

**Affiliations:** ^1^Balance and Dizziness Center, Department of Otorhinolaryngology, Head and Neck Surgery and Audiology, Aalborg University Hospital, Aalborg, Denmark; ^2^Department of Clinical Medicine, Aalborg University, Aalborg, Denmark; ^3^Unit of Clinical Biostatistics and Bioinformatics, Aalborg University Hospital, Aalborg, Denmark

**Keywords:** BPPV, benign paroxysmal positional vertigo, TRV chair, mechanical rotational chair, vertigo, repositioning maneuvers, positional nystagmus

## Abstract

**Background:**

Throughout the last decade, several mechanical rotational chairs have been developed for diagnostics and treatment of patients with a typical case history of benign paroxysmal positional vertigo. Sparse evidence, however, exists in terms of diagnostic accuracy and treatment efficiency with these mechanical rotational chairs. Also, recommendations for optimal use of these chairs are yet to be determined.

**Objective:**

Primary objective was to evaluate overall treatment of benign paroxysmal positional vertigo with a mechanical rotational chair and secondary objectives included description of patient- and BPPV characteristics, determination of subjective and objective outcomes, as well as analyzation of recurrence- and recurrence-related risk factors following successful treatment.

**Methods:**

Retrospective cohort study with 635 patients diagnosed with benign paroxysmal positional vertigo and treated by means of a mechanical rotational chair during a 7-year period from 2014 to 2021 at a tertiary University hospital. Patient- and disease-specific characteristics, treatment and recurrence data were collected through reviewing of patient records.

**Results:**

The mean number of required treatments was 2.7 when accounting for a six percent treatment failure rate (defined as a need of more than 10 treatments), and 3.7 when not. Bilateral mono-canal affection required 3.8 treatments, unilateral multi-canal 3.5 treatments, and the combination of bilateral and multi-canal affection 5.2 treatments. All these scenarios were associated with significantly higher numbers of required treatments when compared to unilateral mono-canal affection, which required 1.9 treatments. The overall recurrence rate was 25.4 percent.

**Conclusion:**

A mechanical rotational chair provides successful treatment of benign paroxysmal positional vertigo. Mechanical rotational chairs should primarily be reserved for the treatment of retractable and atypical benign paroxysmal positional vertigo patients. Many aspects of the optimal use of these chairs still require elaborative assessment.

## Introduction

Benign Paroxysmal Positional Vertigo (BPPV) is a disease characterized by short episodes of vertigo caused by rapid changes of head position (1). Pathophysiologically it is believed that BPPV is caused by otoliths from the utricle which are dislocated into the endolymphatic space in one or several of the semicircular canals (SCCs) within the vestibulum of the inner ear. The debris of the displaced otoliths can either be floating freely (canalolithiasis, CAN) or be attached to the sensory organ (cupulolithiasis, CUP) within the affected SCC(s) (2–4). By far, posterior CAN is the most prevailing subtype of BPPV (85–95%), but the disease entity also includes more complex cases with atypical location, CUP type BPPV, ipsilateral multi-canal affection, or even bilateral affection. The disease is very common and the incidence of BPPV ranges between 11 to 64 per 100,000 per year (1) and the cumulative incidence is almost 10% by the age of 80 (5). An epidemiological study found the lifetime prevalence of BPPV to be 2.4% (5). To a large extent, however, BPPV may recover spontaneously. This happens in ~20–50% of cases after 3 months of follow-up (6–8).

Several treatments have been suggested in order to achieve complete resolution of BPPV. The treatments offered generally consist of conventional repositioning maneuvers (CRMs) that are designed and carried out in a way so certain maneuvers done with the patient on an examination bed clears the SCC by moving the displaced otoliths back into the utricle. Success rates of CRMs have been reported as high as 80–90% (9). The success rate of one of the most widespread CRM, the Epley maneuver, has been reported to be as high as 84% following three or fewer Epley maneuvers (10), and a systematic review from 2014 stated that the odds ratio for complete resolution of vertigo was 4.42 and the odds ratio for conversion of DH from positive to negative was 9.62 with the Epley maneuver (1). If you consider these numbers, however, it becomes evident, that still 10–20% cannot be treated successfully by means of traditional CRMs (11) and are therefore, in this study, considered CRM refractory patients. These patients, who often require multiple treatment visits for relief or develop rapid recurrence of their symptoms, have previously been impossible to treat successfully. It has previously been shown, that two BPPV characteristics have significant impact on the number of treatments required for the relief of symptoms: (1) atypical location (location of BPPV involving any other SCC than a single posterior SCC), and (2) bilateral disease (12).

Because these types of BPPV have been so hard to treat by CRMs, several mechanical rotational chairs (MRCs) have been developed. Potential advantages of MRCs include increased comfort to the patient during diagnostics and treatment, increased convenience, practical to use, increased maneuverability with 360-degree rotation capability in two perpendicular axes, precise orientation, improved safety, all-inclusive nystagmus observation that facilitates improved and more precise diagnostics, and electronic documentation (9, 13). When combining MRC usage with videonystagmography, diagnostic accuracy and repeatability increases because uniform positional testing, independent of the individual examiner, is possible. Concomitant observation, measurement, recording of accompanying positional nystagmus and the subsequent interpretation of this important parameter is thereby optimized by videonystagmography. Finally, MRCs also allows several treatment options that are not possible by means of CRMs (14). Current MRCs include the Thomas Richard-Vitton Repositional Chair^®^ (MRC-1) (Interacoustics, Middelfart, Denmark), the Epley Omniax System^®^ (MRC-2) (Vesticon, Portland, USA), the Rotundum^®^ positioning chair (MRC-3) (Prolim Engineering GmbH, Küsnacht, Switzerland), and the Automated Mechanical Repositioning Treatment (MRC-4) (Byrons Medical Science & Technique Inc., Jinan, China). Few studies have been conducted with the use of the MRC-1, MRC-2 and MRC-4 and, to the knowledge of the authors, several studies with the use of the MRC-1 and MRC-3 are ongoing but have yet not been completed and published.

Previous studies have shown that MRCs are superior especially in the treatment of refractory BPPV or complex BPPV with CUP, ipsilateral multi-canal and bilateral BPPV (14, 15). Treatment of patients with refractory BPPV have also showed significant improvement of subjective outcomes by means of lowering of Dizziness Handicap Inventory-, Visual Analog Scale-, and Hospital Anxiety and Depression Scale scores following treatment with MRCs (16). Treatment of unilateral posterior BPPV with MRCs has also proven superior in terms of fewer treatments needed and by obtaining faster remission initially when comparing treatments with CRM to treatments by MRC (17). It has also been hypothesized that BPPV due to displacement of very small/low density otoliths are more easily cured by means of MRC compared to CRM (18).

Comparisons between different BPPV studies are difficult because no consensus exists in terms of a clear uniform definition of successful BPPV treatment. Important parameters include both the degree of remission of (1) subjective symptoms (positional vertigo) and (2) objective findings (positional nystagmus). With treatment of BPPV patients, several different outcome scenarios may be observed. To display the heterogeneity of this, and in order to enable the reader to choose between different individually preferred outcome parameters, several different ways of defining “successful treatment” have been included.

The primary aim of this study was to evaluate the effectiveness of BPPV treatment with the MRC-1 in a tertiary clinical setting with primarily CRM refractory patients in terms of number of required treatments as well as determination of the overall success rate. Secondary aims included (1) description of patient- and BPPV characteristics with a tertiary setting cohort, (2) determination of subjective and objective outcomes in terms of treatment success rates, and (3) Analyzation of recurrence- and recurrence-related risk factors following successful MRC-1 treatment.

## Materials and methods

The study was designed as a retrospective cohort study with patients who were diagnosed with BPPV and commenced treatment with the MRC-1 during a 7-year period from June 2014 to June 2021 at the tertiary Balance & Dizziness Center, Aalborg University Hospital. Patient and BPPV characteristics, treatment and recurrence data were collected through patient record reviewing. The Research Electronic Data Capture (RedCap^®^) system was used for data storage and handling.

Demographics included sex and age at inclusion. Symptom debut was registered as precis as possible. Midpoints of date intervals was registered, if patients only recollected a broader period, e.g., the 15th of a given month.

Diagnostics were done by a combination of the MRC-1, videonystagmography with the video Frenzel goggles (VF405^®^, Interacoustics, Middelfart, Denmark) and accompanying VisualEyes^tm^ software (Interacoustics, Middelfart, Denmark). The positional nystagmus was quantified in terms of direction (horizontal and/or vertical), number of beats and average slow-phase velocity (a-SPV). Dix-Hallpike (DH) test and Supine Roll Test (SRT) were carried out routinely at every visit throughout the inclusion period. BPPV diagnostics was based upon international standards (19–21). The type of BPPV was defined as the specific combination of the involved SCC(s), side of affection and the location within the SCC(s) (CAN or CUP). BPPV characteristics were categorized as unilateral, bilateral, mono-canal or multi-canal affection.

Treatments were done following completion of the diagnostic tests, and the number of treatments in one treatment session was, with the vast majority of cases, limited to one. The treatment protocols used with every patient varied during the treatment period, but the different repositioning maneuvers that were used are explained in Table 1. Because of the lack of evidence supporting post-treatment restrictions (22, 23), post-treatment restrictions were limited to only avoidance of head down positioning before going to bed in the evening of the day of treatment. Patients were routinely scheduled for a post-treatment follow-up despite any apparent effect of the treatment. The time interval between follow-up sessions varied amongst patients but was normally 1–4 weeks. A tertiary treatment center entails that the majority of the patients had been treated with CRMs prior to referral. However, occasionally the Balance & Dizziness center accepted direct GP referrals due to additional BPPV studies. The proportion of patients who had received CRM treatment prior to referral is shown in Table 2. These patients were categorized as CRM refractory patients as referrals predominantly included CRM treatment resistant BPPV patients or patients with recurring BPPV with short time intervals. The preceding BPPV treatment maneuver(s) or number of treatments prior to referral were not registered, as this information was often missing due to either poor memory or understanding of previous treatments by the patients or due to a low degree of detail with the referrals.

**Table 1:** Treatments offered with the MRC-1 chair.

**Repositioning maneuver**	**Maneuver description**	**BPPV subtype eligible for this treatment**
Modified Epley maneuver	Positioned with the head 30–45° below horizontal level and body (head) rotated 45° toward the affected side (DH position). Followed by four consecutive 45° rotations toward the healthy side with a total of a 180° rotation toward the healthy side. Every position was kept for 30–60 s or until positional nystagmus disappeared.	Posterior CAN
Potentiated Epley maneuver	Same approach as the modified Epley, but 10 impulses, adding kinetic force, were applied in addition in all five positions. Impulses were added by bumping the seat vertically into an attached shock absorber 45° below horizontal level.	Posterior CAN and CUP
Semont maneuver	Same starting position as the modified Epley, but instead of consecutive rotations in the yaw axis (minutes), the patient is rotated 270° in the pitch axis in one fast (few seconds) turn with an abrupt stop against the attached shock absorber on the opposite side. Can be performed with or without impulses in the starting position.	Posterior CAN and CUP
Shock treatment for lateral BPPV^*^	For treatment of lateral CAN begin with body (head) rotated 45° toward the affected side and the body positioned horizontally. Ten impulses are applied in this position and treatment is continued by four consecutive 45° rotations toward the healthy side with impulses (kinetic energy) added in each position. For treatment of lateral CUP the same approach is used. However, treatment of lateral CUP is initiated with the body (head) rotated an additional 45° toward the affected side.	Lateral CAN and CUP
Dynamic barbeque roll	Supine position followed by a 90° rotation toward the affected side. The patient is rotated 360° ten times toward the healthy side with accelerations and deaccelerations included in every rotation.	Lateral CAN and CUP
Deep head hanging maneuver	Body (head) rotated 45° toward the un-affected side. Then a fast forward or backward 180° rotation in the pitch axis is performed. Can be performed with or without impulses before 180° rotation. The maneuver can also be done without any 45° rotation before the 180° rotation in the pitch axis if treatment is intended for patients without unambiguous laterality.	Anterior CAN and CUP
Maneuver for treatment resistant BPPV	The patient is placed in the supine position (lateral BPPV) or further 45° downwards in the pitch axis. The patient is then rotated 135° toward the affected side in the yaw axis. Following treatment in this position the patient is rotated toward the healthy side seven times with 45° intervals. In all eight positions 20 impulses are applied	Posterior and lateral, CAN and CUP
Individualized maneuver	Fewer or more rotational steps and/or impulses with the Epley maneuver or shock treatment due to patient related conditions.	Posterior and lateral, CAN and CUP

* Performed in supine horizontal positioning from June 2019 and onwards. CAN, canalolithiasis; CUP, cupulolithiasis.

**Table 2:** Overall demographics and clinical features.

Total, *n*	635
Female sex, *n* (%)	427
Age, mean (SD)	64 (16.3)
Symptom duration in months, mean (SD)	18 (50.9)
Symptom duration in months, median	5
Follow-up time in months, mean (SD)	9 (12.8)
Follow-up time in months, median	4
Previously treated for BPPV, *n* (%)	336 (52.9)
Primary etiology, *n* (%)	533 (83.9)
**Secondary etiologies, *n* (%)**	
° Head trauma	50 (7.9)
° Vestibular neuritis	25 (3.9)
° Meniere's disease	12 (1.9)
° Previous ear surgery	10 (1.6)
° Meningitis	1 (0.2)
° Other^*^	4 (0.6)
**BPPV characteristics, *n* (%)**	
° Unilateral mono-canal	326 (51.3)
° Unilateral multi-canal	93 (14.6)
° Bilateral mono-canal	95 (15.0)
° Bilateral multi-canal	121 (19.1)
**BPPV type, *n* (%)**	
° Posterior CAN	592 (93.2)
° Posterior CUP	76 (12.0)
° Lateral CAN	189 (29.8)
° Lateral CUP	248 (39.1)
° Anterior CAN	69 (10.9)
° Anterior CUP	55 (8.7)

* Two cases of sudden deafness with vestibular affection, one case with barotrauma and one case of uncharacteristic inner ear trauma after otoscopy that resulted in a sudden short-lasting loud noise followed by a profound hearing loss and complaints of positional vertigo. CAN, canalolithiasis; CUP, cupulolithiasis.

The first course of treatment was defined as the total number of treatments provided from the first treatment with the MRC-1 until BPPV treatment was concluded to be successful. Patients with recurrence(s) would therefore have an additional “course of treatment” with each recurrence. However, the number of treatments with any recurrence(s) was neither registered nor included in this study. Review of patient records revealed that only a few patients were instructed to try manual treatments at home between MRC-1 treatments (BPPV home exercises) or already did this routinely, e.g., home Epley maneuvers and/or Brandt-Daroff home exercises, as patients referred were perceived as CRM refractory.

No international consensus or definitions exist in regard to “successful treatment of BPPV” and no uniform criteria consider both subjective symptoms and objective findings in the evaluation of BPPV treatment. In this study, we therefore had to clearly define possible outcomes for both subjective symptoms and objective findings. Patients were considered successfully treated and therefore discontinued follow-up in the following circumstances: (1) complete remission of objective findings, (2) improvement of objective findings (lowering of positional nystagmus a-SPV) and complete remission of subjective symptoms, (3) improvement of objective findings (lowering of positional nystagmus a-SPV) and improvement of subjective symptoms in combination with either discontinuation of further follow-up treatments (patient decision) or no further improvement observed at several consecutive follow-ups (months). Complete remission of symptoms was hypothesized more unlikely to happen with patients requiring more than five treatments, and therefore, specification of the subjective and objective outcomes at the time of discontinuation were registered in these cases only. Subjective status was divided into the following four groups: complete remission, improvement, no change and worsening. Objective status was divided into five groups: complete remission, improvement (minimum of 20% a-SPV decrease or improvement in positional nystagmus mentioned by the therapist), no change (<20% a-SPV change), worsening (minimum of 20% a-SPV increase or worsening of positional nystagmus mentioned by the therapist) and incomplete data (a-SPV data missing).

In consistency with a previous MRC-1 study (14), patients who required more than ten treatments were considered treatment resistant and therefore classified as “treatment failures”.

Recurrence was defined as recurrent BPPV following successful treatment(s) with the MRC-1 and included patients with substantial worsening of either subjective symptoms or objective findings in patients who were discontinued without complete objective recovery (minor, non-significant, positional nystagmus). Recurrences were divided into three groups: Ipsilateral recurrence within 6 months, contralateral recurrence within 6 months, and ipsilateral or contralateral after 6 months.

Following successful treatment or discontinuation of treatment, patients could request a follow-up, visit without a new referral, in case they experienced symptoms compatible with a relapse of their BPPV. In case of any relapse beyond this 3-month period, patients had to go through primary or secondary care to receive a new referral to the Balance and Dizziness Center. Patient records showed that patients with recurrences after the 3-month limit were generally re-referred after only a few attempts with CRMs, as treatments offered with the MRC-1 had previously proven superior to manual CRMs.

For statistics, Poisson regression with robust variance estimation was used to compare number of required treatments in subgroups. Otherwise, Kruskal-Wallis was used as a non-parametric test for ordinal data and the eta^2^-test (24) was used to calculate the effect size for significant comparisons. The Pearson's Chi^2^-test was used as a non-parametric test for nominal data, and a Phi effects size was used to evaluate significance when doing multiple comparisons. For all comparisons, a *p*-value below 0.05 was considered significant. Software used: StataCorp. 2021. Stata Statistical Software: Release 17. College Station, TX: StataCorp LLC.

## Results

Six hundred and thirty-five patients were included in this study. The distribution of different patient characteristics is seen in Table 2 and comprises the overall findings throughout the follow-up period. The gender ratio was female 2.1:1. Age ranged from 15 to 98 years. BPPV characteristics were compared between subgroups including sex, age, and etiologies, but no significant differences were found.

Table 3 summarizes the findings during the first course of treatment and shows the distribution of the different treatment modalities given.

**Table 3:** Overview of the first course of treatment.

**BPPV characteristics at inclusion, *n* (%)**	
° Unilateral mono-canal	520 (81.9)
° Unilateral multi-canal	57 (9.0)
° Bilateral mono-canal	50 (7.9)
° Bilateral multi-canal	8 (1.3)
**BPPV characteristics based on all sessions during the first course of treatment, *n* (%)**	
° Unilateral mono-canal	369 (58.1)
° Unilateral multi-canal	91 (14.3)
° Bilateral mono-canal	89 (14.0)
° Bilateral multi-canal	86 (13.5)
**BPPV subtypes from all sessions during the first course of treatment, *n* (%)**	
° Posterior CAN	466 (73.4)
° Posterior CUP	57 (9.0)
° Lateral CAN	144 (22.7)
° Lateral CUP	183 (28.8)
° Anterior CAN	47 (7.4)
° Anterior CUP	40 (6.3)
**Treatment modalities used during the first course of treatment, *n* (%)**	
° Epley maneuver	139 (21.9)
° Potentiated Epley maneuver	355 (55.9)
° Semont maneuver	17 (2.7)
° Shock treatment for lateral BPPV	222 (35.0)
° Dynamic barbeque roll	100 (15.7)
° Deep head hanging maneuver	66 (10.4)
° Maneuver for treatment resistant BPPV	5 (0.8)
° Individualized maneuver	72 (11.3)

First course of treatment includes all treatments provided before successful treatment was achieved. Please note that the majority of patients was diagnosed with unilateral mono-canal posterior CAN BPPV, and that the most frequently used treatment modality was the potentiated Epley maneuver. CAN, canalolithiasis; CUP, cupulolithiasis.

For all patients, the cumulative treatment success was 39, 58, 70, and 94% for one, two, three and ten treatments, respectively, while the range for required treatments was 1–55 treatments. For unilateral mono-canal affection the cumulative treatment success was 60, 72, 87, and 98%, respectively, with a range of 1–25 treatments. Forty-five (7%) out of 620 patients, who were successfully treated, required more than 10 treatments and were therefore categorized as treatment failures. When accounting for treatment failure, the overall mean for the number of required treatments to achieve successful treatment was 2.7. If not accounting for treatment failures, the mean number of required treatments was 3.7. Treatment means of subgroups with comparisons are seen in Table 4. BPPV involving more than one SCC required significantly higher numbers of treatments with increasing numbers of required treatments in regard to increasing numbers of SCCs being affected. In relation to BPPV subtypes, CUP required more treatments than CAN. Previous ear surgery (4.6), meningitis (2.0) and other secondary etiologies (5.5) were all significantly different from primary BPPV, but all had sample sizes below ten. There was no difference in treatment requirements between sexes (2.7 vs. 2.7), but with advancing age, treatment requirements increased by 0.5% per year.

**Table 4:** Number of required treatments.

**Subgroup**	***N***	**Mean**	**Relative mean**	**CI 95%**	***P*-value**
All	575^**^	2.7			
Sex					
° Male	190	2.73	1.000		
° Female	385	2.67	0.973	0.81–1.17	0.773
Age at first visit			1.005	1.00–1.01	0.037^*^^‡^
Symptom duration (months)			1.002	1.00–1.004	0.118
Etiology					
° Primary	484	2.64	1.000		
° Secondary	91	2.96	1.156	0.91–1.47	0.239
° Head trauma	45	2.60	0.981	0.69–1.34	0.915
° Vestibular neuritis	21	2.33	0.844	0.48–1.49	0.557
° Meniere's disease	11	3.46	1.391	0.79–2.46	0.256
° Previous ear surgery	9	4.56	1.900	1.10–3.28	0.021^*^
° Meningitis	1	2.00	0.664	0.61–0.73	0.000^*^
° Other	4	5.50	2.364	1.31–4.27	0.004^*^
BPPV characteristics					
° Unilateral mono-canal	354	1.85	1.000		
° Unilateral multi-canal	82	3.45	2.400	1.91–3.02	0.000^*^
° Bilateral mono-canal	80	3.75	2.639	2.13–3.27	0.000^*^
° Bilateral multi-canal	59	*5.22*	3.837	3.13–4.71	0.000^*^
BPPV unilateral mono-canal subtype					
° Posterior CAN (no)	127	2.18	1.000		
° Posterior CAN (yes)	227	1.67	0.614	0.45–0.84	0.002^*^
° Posterior CUP (no)	333	1.80	1.000		
° Posterior CUP (yes)	21	2.62	1.799	1.12–2.81	0.010^*^
° Lateral CAN (no)	309	1.89	1.000		
° Lateral CAN (yes)	45	1.60	0.714	0.46–1.12	0.141
° Lateral CUP (no)	307	1.72	1.000		
° Lateral CUP (yes)	47	2.70	2.056	1.45–2.92	0.000^*^
° Anterior CAN (no)	340	1.86	1.000		
° Anterior CAN (yes)	14	1.71	0.856	1.37–3.54	0.001^*^
° Anterior CUP (no)	341	1.80	1.000		
° Anterior CUP (yes)	13	*3.01*	2.200	1.37–3.54	0.001^*^

At the bottom of the table, all unilateral mono-canal BPPV patients (anterior, lateral, posterior) with either BPPV subtypes [canalolithiasis (CAN) or cupulolithiasis (CUP)] were compared (one-by-one) to the remaining group of patients with unilateral mono-canal BPPV. The group with unilateral mono-canal consisted of 354 patients in total. Please note that posterior CAN and anterior CAN required significantly **lower numbers of treatment** and that posterior CUP, lateral CUP, and anterior CUP required significantly **higher numbers of treatment** when comparing to the remaining group of patients. Please also note that patients with bilateral multi-canal affection and anterior CUP had a mean of more than five and more than three required treatments, respectively. These BPPV patients are therefore the two subgroups with the worst prognosis in terms of expected number of required treatments (shown in italics).

‡ The number of required treatments increased 0.5 percent for every year difference between two respective groups.

* significant, p < 0.05 (all shown in bold).

** 15 out of 635 patients (2%) had ongoing treatment or were lost to follow-up, and 45 patients (14%) were classified as treatment failures.

The subjective and objective outcomes of patients requiring more than five treatments are shown in Table 5 and shows that two thirds of these patients experienced complete subjective remission of vertiginous symptoms and that at least 42% still had some degree of positional nystagmus when discontinued for further follow-ups and treatments.

**Table 5:** Outcomes with patients requiring more than five treatments.

Total number of patients, *n* (%)	102 (16.6^*^)
**Subjective outcome**, ***n*** **(%)**	
° Complete remission	68 (66.7)
° Improvement	30 (29.4)
° No improvement	4 (3.9)
**Objective outcome**, ***n*** **(%)**	
° No positional nystagmus	31 (30.4)
° Less positional nystagmus	40 (39.2)
° Unchanged positional nystagmus	3 (2.9)
° Incomplete data	28 (27.5)
**Decisionmaker when treatment was discontinued**	
° Examiner	78 (77.2)
° Patient^**^	23 (22.8)
**Reason for discontinuation by the examiner**	
° Complete treatment success	70 (89.7)
° No further effect of treatments	8 (10.3)

With the group of patients that required more than five treatments, 98 patients (96.1%) experienced improvement of subjective symptoms and 71 patients (69.6%) had improvement of objective findings.

* 22 out of 635 (3.5%) patients had ongoing treatment or were lost to follow-up at the time of data collection.

** All patients decided to discontinue treatments due to satisfactory subjective treatment results.

The overall recurrence rate was 25% and only 28% of these happened 6 months or more after successful treatment as seen in Table 6. Overall, the mean time from successful treatment until first recurrence, if any, was 172 days. Risk factors associated with recurrences revealed no significant differences with sex and age. However, a tendency (*p* < 0.1) of higher recurrence rates in females was found (28 vs. 21%). Higher numbers of required treatments and bilateral multi-canal affection were positively correlated with higher recurrence rates, while unilateral mono-canal affection was significantly associated with a lower risk of recurrence.

**Table 6:** Recurrence of BPPV and risk factors associated with BPPV recurrences.

**Subgroup**		***P*-value**
Overall number of recurrences, *n* (%)	148 (25.4)^**^	
Number of ipsilateral recurrences within initial 6 months, *n* (%)	88 (15.1)	
Number of contralateral recurrences within initial 6 months, *n* (%)	19 (3.3)	
Number of recurrences after 6 months or later, *n* (%)	41 (7.0)	
**Time from remission to recurrence, median in days (IQR)**		
° All recurrences	172 (90–465)	
° Ipsilateral initial 6 months	95 (55–147)	
° Contralateral initial 6 months	138 (92–159)	
° Recurrences after 6 months or later	515 (312–838)	
Sex, recurrences *n* (%)		0.07
° Male	40 (20.8)	
° Female	108 (27.7)	
Age, median (IQR)		0.58
° BPPV recurrence	66 (53–75)	
° No BPPV recurrence	67 (54–76)	
Number of required treatments, median (IQR)		**0.02** ^*^ ^‡^
° BPPV recurrence	3 (1–4)	
° No BPPV recurrence	2 (1–4)	
Etiology, recurrence *n* (%)		0.82
° Primary	124 (25.3)	
° Secondary	24 (26.4)	
BPPV characteristics, recurrences *n* (%)		**0.00** ^*^ ^Φ^
° Unilateral mono-canal	34 (11.3)	**0.00** ^*^
° Unilateral multi-canal	26 (29.9)	0.30
° Bilateral mono-canal	24 (28.6)	0.47
° Bilateral multi-canal	64 (57.7)	**0.00** ^*^

* Significant, p < 0.05 (shown in bold).

** Based upon 582 patients with a minimum of 6 months follow-up.

‡ The Eta^2^ effect size was 0.008.

Φ Post-hoc analysis with multiple comparisons showed that only unilateral mono-canal compared to bilateral multi-canal had a significant difference plus a Phi effect size of more than 0.3 (0.48). BPPV, Benign paroxysmal positional vertigo; IQR, interquartile range.

## Discussion

The success rate varies from 66 to 100% with the existing literature regarding treatment by means of MRCs (25), but the inter-cohort differences vary greatly in terms of BPPV location, -subtypes, and -characteristics. More importantly, though, the origin of the individual referrals is crucial as some referrals include vertiginous patients for primary diagnostics and treatment and others include CRM refractory cases referred from other secondary ENT centers. Four studies included refractory BPPV patients solely or partially, of which two had no treatment failure cases (16, 26), another study had a 7% failure rate (14) and the last study found a 3% failure rate and a 28% improvement rate (15). Our study showed an overall 94% treatment success rate with a treatment failure definition of 10 or more required treatments. When looking deeper into the subjective and objective outcomes, in patients without any limitations in the number of treatments offered, a larger proportion of patients requiring more than five treatments experienced complete recovery subjectively compared to complete nystagmus remission. This might indicate that, for some patients, the presence of minor otolithic debris may not cause vertigo or that habitualization might facilitate subclinical BPPV. Residual dizziness is known to be present in up to more than half of all patients following successful repositioning and resolves in 95% of cases within 46 days following treatment (27). As most of the patients included in this study had follow-ups scheduled within a few weeks, it seems reasonable to conclude that a large proportion of the patients, who experienced subjective improvement, also were successfully treated objectively and would be completely relieved of vertiginous symptoms eventually.

The overall mean of required treatments with this study was 2.7. In comparison to other similar studies, this is in the upper range of previous studies where a range of 1.5–3 is seen (14–16, 26, 28–30). However, the only two studies with a mean below 2.0 had no multi-canal BPPV patients and one of them only included posterior SCC BPPV (28, 30). Thus, being more comparable to the unilateral mono-canal BPPV group of this study, which had a mean of 1.9 required treatments. Not surprisingly, bilateral- or multi-canal SCC affection required significantly higher numbers of treatments than unilateral mono-canal BPPV, and the combination of these two groups had a mean of 5.2 required treatments. Also, all CUP BPPV subtypes required significantly higher numbers of treatments. This is in concordance with other studies (9, 14, 15, 26).

A recent study compared treatment with the MRC-1 with CRM in non-refractory BPPV patients (31). That study found no significant differences in required numbers of treatments between the two groups. However, a tendency of higher numbers of required treatments for non-posterior BPPV was observed in the CRM group. Another study compared treatments with the MRC-1 to CRM treatments in non-refractory posterior BPPV patients and found a significant short-term favor of treatments provided by the MRC-1 (17). A third study compared CRM treatments with MRC-4 treatments in patients diagnosed with posterior BPPV and found that the MRC-4 required significantly less treatments (1.5 vs. 1.9) than CRM (30).

The proportion of atypical BPPV patients is very high within this study population, where 42% of the first course BPPV characteristics include bilateral or multi-canal affection BPPV patients and 30% of all type CUP subtype BPPV patients. These percentages of atypical BPPV cases are substantially higher when comparing to similar studies from other tertiary centers where multi-canal patients, including bilateral BPPV patients, ranged from 5 to 27% and where CUP subtype BPPV patients ranged from 5 to 39% (9, 12, 15, 26). The proportion of bilateral BPPV patients in this study was 28 and 32% in a similar study (29), but these numbers are likely overestimations, as determination of lateralization of horizontal canal BPPV remains unclear in up to 20% of patients (19, 28). The Bow and Lean test (32) may be added to improve the diagnostics in order to determine laterality of horizontal BPPV but, despite addition of this test, determination of laterality may prove impossible. The overall BPPV recurrence rate in our study was 25% with 72% occurring within the initial 6 months of follow-up. Without doubt, this is an underestimation of the true recurrence rate of our cohort due to both the retrospective nature of this study as well as the 3 months window post treatment before a new referral was required. BPPV recurrence rates in previous studies still, to a large extent, vary significantly, and many studies are limited by retrospective designs or short follow-up periods. One recent review found a range in recurrences between 13 and 68% in studies with a follow-up period of more than 24 months (33). However, this review also included retrospective studies. Prospective studies, surprisingly, also report different recurrence rates despite several years of follow-up. The range of BPPV recurrence rates was 18–67% in six prospective studies with at least 2 years of follow-up and showed an average BPPV recurrence rate of 34%. Recurrence rates of two of these studies, with an average follow-up period of 10 years, still varied more than 30% (16, 34–38). However, it seems to be consistent throughout the literature, including this study, that the majority of BPPV recurrences happen within the first six months following successful repositioning. It also seems that BPPV recurrences are rare more than 2 years after successful treatment (34, 36, 37).

High number of required treatments, as well as bilateral and multi-canal disease, were significantly associated with an increased risk of BPPV recurrences. Bilateral and multi-canal disease were also proven to contain more intractable BPPV characteristics and therefore also confounding factors in the correlation between numbers of treatments required to treat successfully as well as frequency of BPPV recurrences. Age and sex were not significantly associated with higher recurrence rates, though female sex had a tendency (*p* < 0.1). Existing literature find contradictory results in regard to sex and age, as some find female sex and advanced age as risk factors (36, 39, 40), while others do not (34, 37, 41, 42). Other risk factors include hypertension, hyperlipidemia, vitamin D deficiency and osteoporosis (34, 41, 42), which are also known risk factors of BPPV occurrence (43). Our study found no difference in BPPV recurrence rates between primary and secondary etiologies. Other studies have assessed individual secondary etiologies, but there are found no consistent findings (39–42).

Several aspects of treatment with the MRC-1 are still dependent on the individual therapist although the MRC-1 is designed with several predefined positions. These non-standardized aspects include duration and velocity between positions, time that each position is maintained, as well as the energy induced by each individual impulse. Therefore, the number of required treatments may differ significantly because of inter-therapist differences that are not clear with this study.

The MRC-1 comes without a detailed or evidence based manual for BPPV diagnostics and treatment. This makes inter study comparisons very difficult because several elements of diagnostics and treatments might differ significantly between individual therapists. However, diagnostics and treatments with the MRC-1 at our clinic is standardized by means of a continuously updated local protocol that, to a certain extent, allow the individual therapist to choose a specific treatment modality with individualized adaptation based upon their clinical experience.

In general, prerequisites for optimal usage of the MRC-1 chair include preexisting knowledge and understanding of the BPPV disease entity as well as previous experience with traditional BPPV diagnostics and treatments to be able to transfer the clinical findings during examination and treatment into theoretical otolith location and behavior. Treatment success may therefore also rely upon the individual therapist's pre-existing BPPV knowledge and previous clinical experience with usage of the MRC-1.

Due to the retrospective nature of the study, the objective outcome measures with the patients that required more than five treatments were limited to the a-SPV or the, to a certain degree subjective, nystagmus observation of the therapist. Optimal protocols in a prospective study should exclusively include unambiguous objective measurements and should also include mandatory diagnostic BPPV testing at the last follow-up-even despite subjective reporting of complete remission of vertiginous symptoms. With multi-canal BPPV patients, repositioning of one SCC before treatment of another SCC should also be a prerequisite. Besides registering individual a-SPV values for horizontal, vertical and/or torsional nystagmus, objective measurements could also include additional parameters including a description of latency and duration of the positional nystagmus.

## Conclusion

With CRM refractory BPPV patients, the MRC-1 provides successful treatment. Results support repetitive and numerous treatments as successful treatment is possible even after ten treatments. However, with these atypical cases, re-evaluation of the BPPV diagnosis and/or BPPV subtype should be considered. Evaluation of clinical treatment success should not solely be based upon objective findings of positional nystagmus, as patients may be relieved of their positional vertigo despite minor degree of residual positional nystagmus. Many aspects of the use of MRCs are still to be further assessed, and future studies should especially focus on possible improvements of diagnostics, treatment of CRM refractory patients, optimal treatment procedures as well as cost-benefit analyses.

## Data availability statement

The raw data supporting the conclusions of this article will be made available by the authors, without undue reservation.

## Ethics statement

Ethical review and approval was not required for the study on human participants in accordance with the local legislation and institutional requirements. Written informed consent for participation was not required for this study in accordance with the national legislation and the institutional requirements.

## Author contributions

Concept: DH, SV, and MT. Design, supervision, and literature search: DH and MT. Resources: DH, SV, MB, and MT. Materials, data collection, processing, analysis, interpretation, writing, and critical reviews: DH, SV, NB, MB, and MT. All authors contributed to the article and approved the submitted version.

## Conflict of interest

The authors declare that the research was conducted in the absence of any commercial or financial relationships that could be construed as a potential conflict of interest.

## Publisher's note

All claims expressed in this article are solely those of the authors and do not necessarily represent those of their affiliated organizations, or those of the publisher, the editors and the reviewers. Any product that may be evaluated in this article, or claim that may be made by its manufacturer, is not guaranteed or endorsed by the publisher.
